# Isolated angioedema of the bowel due to C1 esterase inhibitor deficiency: a case report and review of literature

**DOI:** 10.1186/1752-1947-5-62

**Published:** 2011-02-14

**Authors:** Shivangi T Kothari, Anish M Shah, Deviprasad Botu, Robert Spira, Robert Greenblatt, Joseph Depasquale

**Affiliations:** 1 Department of Gastroenterology, School of Health and Medical Sciences Seton Hall University, South Orange, NJ, USA; 2 Department of Internal Medicine, Trinitas Hospital, NJ, USA; 3 Department of Gastroenterology, Trinitas Hospital, NJ, USA

## Abstract

**Introduction:**

We report a rare, classic case of isolated angioedema of the bowel due to C1-esterase inhibitor deficiency. It is a rare presentation and very few cases have been reported worldwide. Angioedema has been classified into three categories.

**Case presentation:**

A 66-year-old Caucasian man presented with a ten-month history of episodic severe cramping abdominal pain, associated with loose stools. A colonoscopy performed during an acute attack revealed nonspecific colitis. Computed tomography of the abdomen performed at the same time showed a thickened small bowel and ascending colon with a moderate amount of free fluid in the abdomen. Levels of C4 (< 8 mg/dL; reference range 15 to 50 mg/dL), CH50 (< 10 U/mL; reference range 29 to 45 U/ml) and C1 inhibitor (< 4 mg/dL; reference range 14 to 30 mg/dL) were all low, supporting a diagnosis of acquired angioedema with isolated bowel involvement. Our patient's symptoms improved with antihistamine and supportive treatment.

**Conclusion:**

In addition to a detailed comprehensive medical history, laboratory data and imaging studies are required to confirm a diagnosis of angioedema due to C1 esterase inhibitor deficiency.

## Introduction

The term 'angioedema' describes a circumscribed edema of the skin, gastrointestinal (GI) tract or respiratory tract. Classic hereditary angioedema (HAE) can be associated with quantitative (type I) or qualitative (type II) deficiency of C1 esterase inhibitor (C1-INH), which is caused by mutations in the *C1-INH *gene [1].

In classic HAE, abdominal attacks are mostly characterized by pain, vomiting and diarrhea, but rarely occur in the absence of other clinical features. These symptoms are due to transient edema of the bowel wall, which can lead to intestinal obstruction, ascites and hemoconcentration. The diagnosis is based on the history of attacks and is confirmed by laboratory testing of C4 levels along with antigenic and functional C1-INH levels.

We describe a case of isolated angioedema of the bowel, a rare presentation, occurring in a man with C1-INH deficiency [2,3]. We review the various types of angioedema, their diagnosis, clinical features and treatment, with emphasis on the GI features and the management.

## Case presentation

A 66-year-old Caucasian man presented with a 10-month history of episodic severe cramping abdominal pain, associated with loose stools. Each episode lasted for a few days and would resolve spontaneously. The patient's medical history included renal cell carcinoma and nephrectomy, transurethral resection of the prostate for benign prostatic hyperplasia, and hypertension, He denied any use of non-steroidal anti-inflammatory drugs or angiotensin-converting enzyme (ACE) inhibitors, any history of urticaria or laryngeal edema, any drug allergies, or any family history of angioedema. He had not recently changed his diet or started new medication.

**Figure 1:**
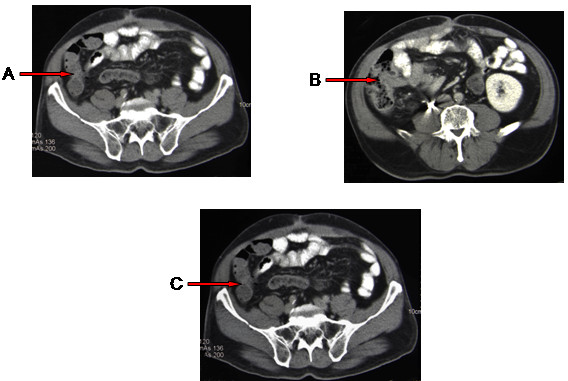
**A-C) Computed tomography scan of abdomen with contrast shows normal appearing small bowel and colon during the asymptomatic phase**.

We gave our patient an extensive evaluation, which included several esophagogastroduodenoscopies and colonoscopies over the span of one year, with normal endoscopic findings. Colonoscopies performed between attacks did not show any evidence of inflammation. Histological examination of biopsies did not reveal any atypical cells or expanded collagen bands. Computed tomography (CT) of the abdomen performed when the patient was asymptomatic showed a normal small bowel (Figure 1). A colonoscopy performed during an acute attack revealed non-specific colitis, and CT of the abdomen performed at the same time showed a thickened small bowel and ascending colon with a moderate amount of free fluid in the abdomen. Abdominal arteriography showed a patent celiac artery and superior mesenteric artery (SMA).

The surgical department was consulted and patient underwent an exploratory laparatomy. An appendectomy was performed and a cecal biopsy obtained, which was normal. However, our patient continued to have similar attacks.

A small bowel series was performed, and showed mucosal irregularities and intramural edema of the distal ileum. Our patient was hospitalized and treated with intravenous hydration. He was afebrile at this time. Laboratory investigations revealed that his white blood cell count was 12.7 × 10^9^/L with 89.4% polymorphonuclear lymphocytes (reference range 4 to 11 × 10^9^/L and 41.5% to 65%). Haemoglobin was 17 g/dL (reference range 13 to 18.0 g/dL) consistent with hemoconcentration, and the chloride was low at 99 mmol/L (reference range 95 to 107 mmol/L). Results of liver function tests and levels of amylase and lipase were within normal limits.

**Figure 2:**
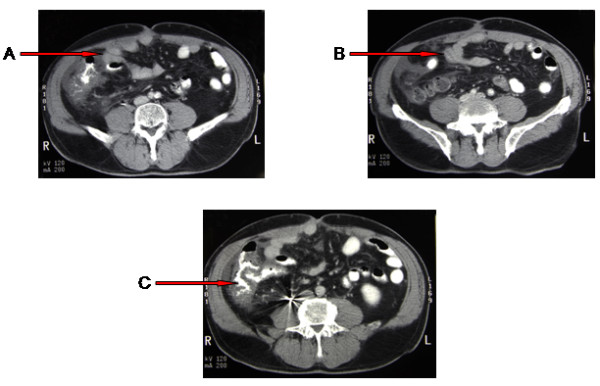
**A-C) Computed tomography with contrast shows extensive concentric colonic and small bowel thickening with trace ascites**.

A repeat inpatient CT scan showed extensive concentric right and transverse colon thickening, and concentric thickening of several small bowel loops with ascites (Figure 2). CT angiography of the abdomen showed the previous finding of a patent celiac artery and SMA.

Further laboratory analysis revealed a normal erythrocyte sedimentation rate. Stool analysis gave negative results for *Yersinia*, ova and parasite, and standard culture. Levels of prostate specific antigen, carcinoembryonic antigen, anti-neutrophil cytoplasmic antibody, anti-*Saccharomyces cerevisiae *antibody, methemoglobin level, and urine porphobilinogen levels were within normal limits. Celiac serology testing and testing for anti-nuclear antibody and carbon monoxide levels were negative. C3 levels were within normal limits. Levels of C4 (< 8 mg/dL; reference range 15 to 50 mg/dL), CH50 (< 10 U/mL; reference range 29 to 45 U/ml) and C1 inhibitor (< 4 mg/dL; reference range 14 to 30 mg/dL) were all low, supporting a diagnosis of acquired angioedema (AAE) with isolated bowel involvement. It is possible, although rare, for a *de novo *genetic mutation to lead to angioedema.

Our patient's symptoms improved with antihistamine and supportive treatment, with danazol treatment planned on an outpatient basis for prophylaxis after discharge. However, our patient had no recurrences of similar episodes during follow-up after discharge and we decided not to start him on any prophylaxis. He has had no attacks since discharge.

## Discussion

**Figure 3:**
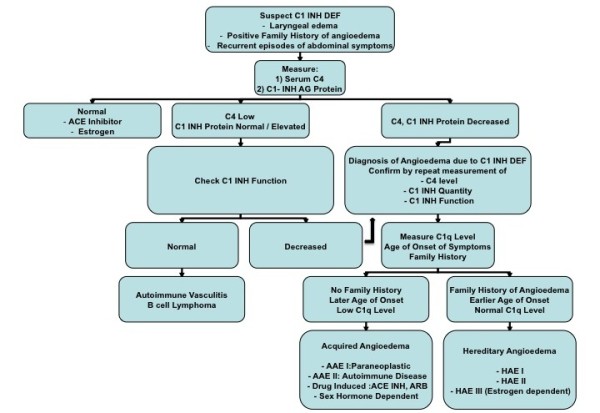
**Diagnostic approach to C1 inhibitor deficiency**.

Angioedema is characterized by marked swelling, which can involve the skin, GI tract and other organs. It has been classified into three categories (Figure 3, Table 1): HAE, AAE and idiopathic angioedema (IAE) [4].

**Table 1:** Classification of angioedema

Hereditary	Acquired	Idiopathic
HAE-I	AAE-I	Nonhistaminergic

HAE-II	AAE-II	

Estrogen dependent (HAE-III)	Sex hormone dependent	

	Drug induced (ACE-I, ARB)	

HAE = hereditary angioedema, AAE = acquired angioedema, ACE = angiotensin converting enzyme, ARB = angiotensin receptor blocker.

HAE was first described by Quincke in 1882 [5]. In 1888, Osler [6] documented its hereditary nature, which was further defined as autosomal dominant by Crowder and Crowder in 1917. HAE type I with plasma protein C1 inhibitor defect was first described by Donaldson in 1963 [6]. The incidence of HAE is estimated at 1 in 50,000. No gender or ethnic group differences have been noted [4]. Symptoms typically worsen after puberty; however, there are reports in the literature of patients developing HAE as late as the ninth decade of life. The severity of the disease usually improves in the seventh and eighth decades of life [7,8]. A nonhistaminergic form of angioedema occurs in about 1 in 20 cases. It is usually not responsive to antihistamines and not associated with urticaria [3]. HAE is divided into 3 types: (1) HAE-I, caused by a *C1-INH *gene mutation resulting in low levels or absence of antigenic and functional C1-INH; (2) HAE-II, caused by a *C1-INH *gene mutation resulting in a normal or high C1-INH antigen level but reduced enzymatic activity and a low functional C1-INH level; (3) estrogen-dependent HAE, which has normal C1-INH levels and function and normal genetic analysis, but a direct correlation with estrogen levels.

AAE was not identified until 1972 [7]. This form is usually associated with autoimmune disease, paraneoplastic syndromes, medications such as ACE inhibitors or changes in estrogen levels. There is no familial inheritance, and it can occur at a later age than HAE. The condition develops as a result of increased consumption of C1-INH or impairment of C1-INH function due to autoantibody formation. Acquired angioedema (AAE) is divided into four groups: (1) AAE-I, resulting from paraneoplastic syndrome or development of autoantibodies which enhances cleavage of C1-INH, leading to C1-INH dysfunction; (2) AAE-II, resulting from autoimmune disease; (3) AAE associated with sex hormones, especially in pregnancy; and (4) drug-induced AAE, particular associated with ACE inhibitors or angiotensin receptor blockers.

IAE is usually associated with urticaria; thyroid dysfunction is common with this form and testing should be performed for it. The mechanism of this disease is unknown.

Angioedema is believed to be due to increased levels of bradykinin associated with C1-INH deficiency. C1-INH is a member of the serpin family of serine protease inhibitors. It is produced mainly in the parenchymal cells of the liver, and is also found in microglial cells, fibroblasts and endothelial cells. It controls the four different enzymatic cascades: the complement system, coagulation, fibrinolytic, and kinin system cascades [4,9]. C1-INH inhibits the formation of activated C1 and the cleavage of C2 and C4. It also inhibits the ability of plasmin to activate C1 and to generate bradykinin from C2. Other protease inhibitors such as antithrombin III, β2-macroglobulin, α1-antitrypsin and α2-antiplasmin also inhibit the ability of plasmin to generate bradykinin, suggesting options for potential therapeutic interventions. The classic presentation of angioedema includes the most common symptom, difficulty in breathing due to laryngeal edema, along with edema of the skin and severe episodic abdominal pain.

Two types of abdominal attacks have been described; the upper GI and the lower GI types. The upper GI type is extremely painful and associated with nausea, vomiting and hypotension without any diarrhea. The lower GI type is accompanied by diarrhea without vomiting [1].

A recent retrospective study involving 153 patients with HAE reported abdominal pain attacks in five phases. Phase I starts with a period of non-cramping abdominal discomfort followed by (phase II) a crescendo phase which leads to (phase III) severe pain. Phase III is associated with vomiting and occasional diarrhea. Hypovolemia and hemoconcentration can occur as a result of a combination of events including vasodilatation, fluid shifts with edema of the bowel, ascites, and volume depletion related to vomiting and diarrhea. Phase IV refers to a decrescendo phase, which is a self limiting phase for untreated abdominal pain. Phase V refers to the resolution of pain, which can occur as often as twice a week [1]. Rare symptoms including hemorrhagic stools, tetany, intussusception and dysuria have been reported.

The diagnosis of angioedema is made in the context of the appropriate constellation of medical history and clinical findings along with supportive laboratory data. HAE should be suspected when there is a history of recurrent angioedema without urticaria and an early age of onset. Symptoms typically worsen over 24 hours and subside in 48 to 72 hours. Swelling of the upper airways, skin or GI tract triggered by minor trauma or a stressor, which fails to respond to treatment with epinephrine, antihistamines or corticosteroids should also raise the index of suspicion for angioedema. Patients suspected of having angioedema should be screened by measuring C4 levels, which are typically low except between the episodic phases. The most sensitive and confirmatory test for detection of HAE is the measurement of C4d, a cleavage product of C4, which is abnormal in patients with HAE even when the C4 level is normal. If the C4 level is decreased, antigenic and functional levels of C1-INH should be measured to confirm the diagnosis.

There are several criteria for diagnosis of angioedema. The major clinical criteria are: (1) self-limiting, recurrent angioedema without urticaria, lasting for more than 12 hours; (2) recurrent self-limiting abdominal pain, lasting for more than six hours or (3) recurrent laryngeal edema. A minor criterion is a family history of recurrent laryngeal edema, angioedema or abdominal pain. Laboratory criteria are (1) antigenic C1-INH levels < 50% of normal at two separate determinations with the patient in basal condition and after the first year of life; (2) functional C1-INH levels < 50% of normal at two separate determinations with the patient in basal condition and after the first year of life; or (3) mutation in the *C1-INH *gene altering protein synthesis and or function. The diagnosis can be made confidently in the presence of one major clinical and one laboratory criterion.

It is useful to measure C1q level as this is typically decreased in acquired C1-INH deficiency but normal in HAE (Figure 3). It is not recommended to measure complement C3 and CH50 levels, as they are near normal in angioedema.

Conventional radiographs of abdomen and barium followed through during acute attacks depict multiple dilated small bowel loops with regular thickened mucosal folds. A 'stacked-coin' appearance, 'thumb printing' and wall thickening may be seen. CT of the abdomen may reveal bowel thickening and ascites with fluid accumulation in the colon, regardless of the severity of the attack.

There are three therapeutic goals for patients with angioedema: immediate treatment, short-term prophylaxis and long-term prophylaxis. In acute emergencies such as laryngeal edema, the immediate treatment is to maintain an open airway. Abdominal pain secondary to edema can be controlled with analgesics. Corticosteroids are of little benefit for patients with angioedema. Short term prophylaxis involves the withdrawal of precipitating factors such as oral contraceptives or hormone therapy, as angioedema can worsen with estrogen therapy. Attacks have also been associated with stress. The use of fresh frozen plasma remains controversial as it might provide factors that could worsen the angioedema. Replacement of the missing enzyme as C1-INH concentrate is the ideal therapy as an immediate, short term or long term treatment.

Danazol, an attenuated androgen, is used for both short and long term prophylaxis. For short term prophylaxis, (for example, before surgery or dental procedures), danazol up to 600 mg/day for five days is recommended. For long term prophylaxis, danazol can be used in doses ranging from 50 to 600 mg/day according to two different protocols. The Milan protocol recommends danazol at high doses of 400 to 600 mg for four weeks, with the dosage tapered every four weeks to the minimal effective dose. The Budapest protocol recommends starting at lower doses and increasing the dose if needed [4]. In patients with frequent episodes of angioedema, the Milan protocol is preferred, and for those with mild episodes of angioedema, the Budapest protocol with a low dose is recommended.

Other agents used for acute therapy and short and long term prophylaxis are antifibrinolytic agents such as tranexamic acid and ε-aminocaproic acid. Tranexamic acid reduces the frequency of swelling in 70% of patients. It is used in children with HAE as the first agent of choice to avoid the virilizing effects of androgens, and is also used in pregnancy. Other potential agents under study are a human plasma-derived C1-INH (Berinert P; CSL Behring, Marburg, Germany); icatibant (a specific peptidomimetic bradykinin 2 receptor antagonist), cinnarizine, plasma kallikrein antagonists, bradykinin antagonists, serine protease inhibitors and gene therapy. DX-88 (ecallantide), a 60-amino acid recombinant protein, which is a specific potent inhibitor of human plasma kallikrein, has been used successfully in the treatment of patients experiencing acute HAE attacks. Cinryze, a plasma derived C1-INH, has been recently approved by the FDA for prophylactic use for HAE in the USA. There are isolated case reports suggesting benefit of epinephrine use during acute attack but the pharmacologic effects of epinephrine do not explain the pathophysiological perturbations underlying C1-INH deficiency. A plasma kallikrein 1B antibody (13G11) helps to detect prekallikreins and hence may be a helpful test.

There is a 50% risk of a child inheriting the disease from a parent with angioedema. In the absence of a genetic diagnosis, it is generally acceptable to wait until the child is at least one year of age before measuring the blood for C1-INH defect. As HAE is an autosomal disorder, when a definitive diagnosis is made, early testing of family members is advisable. Affected individuals need to be educated about the disease and its inheritance to enable informed decisions about future family planning.

Close follow-up with appropriate testing is essential for patients with HAE, and it is advisable for them to wear a medical alert product.

## Conclusion

In summary, the diagnosis of angioedema should be considered in any patient with recurrent abdominal pain of obscure origin. There may not be any abnormal findings between attacks, therefore a comprehensive history and physical examination is of the utmost importance. Confirmatory laboratory data should be obtained and imaging studies performed to confirm the diagnosis.

## Consent

Written informed consent was obtained from the patient for publication of this case report and accompanying images. A copy of the written consent is available for review by the Editor-in-Chief of this journal.

## Competing interests

The authors declare that they have no competing interests.

## Authors' contributions

STK was the primary author, and contributed to patient's diagnosis and treatment in addition to collecting patient information, preparing the framework of the manuscript, tables, illustrations, and writing the entire case report with discussion section. AS and DB contributed to the literature review and data collection. JD worked on the image capturing, legend writing, reference searching and literature review. RG was the primary attending physician on this case, and worked on the data collection. RS worked on the case formatting with grammatical checking and verification of the discussion section with references. All authors read and approved the final manuscript.
