# Isolated angioedema of the bowel due to C1 esterase inhibitor deficiency: a case report and review of literature

**DOI:** 10.1186/1752-1947-5-467

**Published:** 2011-09-20

**Authors:** Shivangi T Kothari, Anish M Shah, Deviprasad Botu, Robert Spira, Robert Greenblatt, Joseph Depasquale

**Affiliations:** 1 Department of Gastroenterology, School of Health and Medical Sciences Seton Hall University, South Orange, NJ, USA; 2 Department of Internal Medicine, Trinitas Hospital, NJ, USA; 3 Department of Gastroenterology, Trinitas Hospital, NJ, USA

## Correction

Following the publication of our article [1] an error in the discussion section was noted. In the description of the five phases of abdominal pain attacks associated with classic hereditary angioedema [2], we incorrectly stated the phases and described phase V instead of phase zero.

Phase I starts with a period of non-cramping abdominal discomfort followed by (phase II) a crescendo phase which leads to (phase III) severe pain. Phase III is associated with vomiting and occasional diarrhea. Hypovolemia and hemoconcentration can occur as a result of a combination of events including vasodilatation, fluid shifts with edema of the bowel, ascites, and volume depletion related to vomiting and diarrhea. Phase IV refers to a decrescendo phase, which is a self limiting phase for untreated abdominal pain. Phase V refers to the resolution of pain, which can occur as often as twice a week

### Should read

Phase zero also known as Prephase which includes fatigue, irritability, sensitivity to noise, nausea, and erythema marginatum. Phase I starts with a period of non-cramping abdominal discomfort followed by (phase II) a crescendo phase which leads to (phase III) severe pain. Phase III is associated with vomiting and occasional diarrhea. Hypovolemia and hemoconcentration can occur as a result of a combination of events including vasodilatation, fluid shifts with edema of the bowel, ascites, and volume depletion related to vomiting and diarrhea. Phase IV refers to a decrescendo phase, which is a self limiting phase for untreated abdominal pain
